# Distorted probability operator for dynamic portfolio optimization in times of socio-economic crisis

**DOI:** 10.1007/s10100-022-00834-0

**Published:** 2022-12-09

**Authors:** Kerem Uğurlu, Tomasz Brzeczek

**Affiliations:** 1 Department of Mathematics, Nazarbayev University, Astana, Kazakhstan; 2 Engineering Management Department, Poznan University of Technology, Poznan, Poland

**Keywords:** Probability distortion, Markov decision processes, Dynamic programming, Risk management, Mathematical finance

## Abstract

A robust optimal control of discrete time Markov chains with finite terminal *T* and bounded costs or wealth using probability distortion is studied. The time inconsistency of these distortion operators and hence its lack of dynamic programming are discussed. Due to that, dynamic versions of these operators are introduced, and its availability for dynamic programming is demonstrated. Based on dynamic programming algorithm, existence of the optimal policy is justified and an application of the theory to portfolio optimization along with a numerical study is also presented.

## Introduction

Starting with political and economical crisis in 1990’s and recent global Covid-19 pandemic have caused unstable market periods. Most recently, in the global pandemic, stock prices has witnessed sharp drops and peaks (Batabyal and Killinis 2021). This turmoil event is called a *black swan*. They are said to be unpredictable with mathematical models as being not reflected in historical data (Phillips 2019; Werther 2017). However, for sure monthly expected rate of return for any financial investment in stock is overestimated for a period of data given turbulence will occur (Focard and Fabozzi 2009). Good market returns are biased with overestimation of the probability of positive extremely high returns. To overcome this, using distortion operators is a well known technique to mitigate optimistic expectations. It is used frequently in behavioural finance (see e.g. Kahneman and Tversky 1979, Kahneman and Tversky 1992, Zhou 2010). It has been motivated by empirical studies in behavioural finance and aims to model the human tendency to exaggerate small probabilities of extreme events (Wakker 2010). In particular, it is only natural to consider distortion operators to estimate the risk during global crisis such as the recent pandemic period. One can hinder the error of naively predicting the future returns with a current state of the market that is changing all the time. The distortion operator is shown to be robust to optimistic high returns. Namely, it is more sensitive to the risk of negative returns than the expectation operator by mitigating the chance of positive returns. In particular, the distortion operator is *risk-averse*. It has a lot in common with value investment strategies that are shown to outperform market return-risk efficiency even in long term. This is to be observed since economic turmoil in 2008 (see Elze 2012, Majewski et al. 2020).

In that respect, models with risk aversion has a long history, and they are represented via alternative approaches. One of these approaches is using concave utility functions modelling risk-aversion (see e.g. Chung and Sobel 1987, Fleming and Sheu 1999, Fleming and Sheu 2000, Jaquette 1973, Jaquette 1976) and the references therein), where the utility functions are subject to satisfy some regularity properties. Another approach uses the so-called coherent risk measures introduced in Artzner et al. (1999). Here, these risk measures are again subject to satisfy some axioms modelling the random outcomes taking risk awareness into consideration. We refer the reader to Artzner et al. (2007); Ruszczynski (2010); Cheridito et al. (2006); Eichhorn and Romisch (2005); Follmer and Penner (2006); Fritelli et al. (2002, 2005)) and the references therein for further details on the risk measures.

On the other hand, although modelling random outcomes representing gains/losses using probability distortion goes back to at least 1970’s (Kahneman and Tversky 1979), its axiomatic incorporation into multiperiod settings is still absent in the literature. There are few recent works in this direction. To mention few, (He and Zhou 2011) studies a portfolio optimization problem in continuous time using probability distortion, whereas (Kun et al. 2018) studies a discrete time controlled Markov chain in infinite horizon. In another work, (Ma et al. yyy) assumes monotonicity of the cost/gain functions and presents the results under that assumption. The reason for scarcity of the literature in multiperiod setting lies in the fact that the distortion operator is of limited use in control problems due to not satisfying “Dynamic Programming Principle” (DPP) or “Bellman Optimality Principle”. Namely, a sequence of optimization problems with the corresponding optimal controls is called time-consistent, if the optimal strategies obtained when solving the optimal control problem at time *s* stays optimal when the optimal control problem (OCP) is solved at time $t>s$ (Bjork et al. 2017). OCP’s are of vital importance in various fields of operations research In this paper, we introduce a *dynamic version* of probability distortion that does not suffer from time-inconsistency. DPP can be applied readily in our framework under controlled Markov chains, and additionally DPP gives the existence of the optimal policy.

The rest of the paper is organized as follows. In Sect. 2, we describe probability distortion on random variables in a static one-period case first. Next, we introduce the concept of dynamic probability distortion on stochastic processes in a multi-temporal discrete time setting. In Sect. 3, we introduce the controlled Markov chain framework that we are going to work on. In Sect. 4, we state and solve our optimal control under study, by characterizing both the optimal value and policies as solutions of the dynamic programming equations. In Sect. 5, we illustrate our results on a portfolio optimization problem and conclude the paper.

## Probability distortion

In this section, the concept of probability distortion along with the corresponding operator and its properties are introduced. These definitions are further extended to the multi-temporal/dynamic setting.

### Probability distortion on random variables

Let $(\Omega , \mathcal {F}, \mathbb {P})$ be a probability space and denote by $L^{\infty }_{+}(\Omega ,\mathcal {F},\mathbb {P})$ the set of non-negative essentially bounded random variables on $(\Omega ,\mathcal {F})$.

#### Definition 2.1

- A mapping $w : [0,1] \rightarrow [0,1]$ is called a distortion function, if it is continuous, strictly increasing, and satisfies $w(0) = 0$ and $w(1) = 1$.
- For any $\xi \in L^{\infty }_+(\Omega ,\mathcal {F},\mathbb {P})$, the operator with respect to the distortion function *w* is defined by
  2.1 $$\begin{aligned} \rho (\xi ) \triangleq \int _0^\infty w \left( \mathbb {P}(\xi \ge z )\right) dz \end{aligned}$$

#### Lemma 2.1

(i) Let $x,y, \alpha \in [0,1]$, $\xi \in L^{\infty }_+(\Omega ,\mathcal {F},\mathbb {P})$ and $w:[0,1] \rightarrow [0,1]$ be a distortion function that satisfies
  2.2 $$\begin{aligned} w(\alpha x + (1- \alpha ) y ) \ge \alpha w(x) + (1-\alpha )w(y). \end{aligned}$$
  Then, $\rho (\xi ) \ge \mathbb {E}[\xi ]$. Namely, for $\xi$ representing the nonnegative bounded random losses, $\rho (\cdot )$ evaluates a bigger risk for $\xi$ than $\mathbb {E}[\cdot ]$ does.
(ii) Conversely, suppose *w* satisfies for $\alpha \in [0,1]$
  2.3 $$\begin{aligned} w(\alpha x + (1- \alpha ) y ) \le \alpha w(x) + (1-\alpha )w(y), \end{aligned}$$
  then $\rho (\xi ) \le \mathbb {E}[\xi ]$. Namely, for $\xi$ representing the nonnegative bounded random gains, $\rho (\cdot )$ evaluates a smaller gain for $\xi$ than $\mathbb {E}[\cdot ]$ does.

#### Proof

We will only prove the first part. By $w(0) = 0$ and (2.2), we have $w(\alpha x ) \ge \alpha w(x)$ for any $\alpha \in [0,1]$. In particular, for $x = 1$, we get $w(\alpha ) \ge \alpha$ for any $\alpha \in [0,1]$. Thus, $w(\mathbb {P}(\xi \ge z )) \ge \mathbb {P}(\xi \ge z )$ for any $z \in \mathbb {R}$. By taking integrals on both sides, we conclude the result. $\square$

#### Remark 2.1

Lemma 2.1 implies that (2.2), respectively (2.3), is an appropriate property of the distortion function *w* for modelling risk averse behaviour towards random costs, respectively towards random profits.

#### Lemma 2.2

Let $\rho : L^{\infty }_{+}(\Omega ,\mathcal {F},\mathbb {P})\rightarrow \mathbb {R}$ be the distortion operator as in (2.1). Then

(i) $\rho$ is positively translation invariant, i.e., $\rho (\xi + c) = \rho (\xi ) + c$ for $c \ge 0$. In particular, $\rho (c) = c$ for any $c \ge 0$.
(ii) $\rho$ is positively homogeneous, i.e. $\rho (\lambda \xi ) = \lambda \rho (\xi )$ for $\lambda \ge 0$.
(iii) $\rho$ is monotone, i.e. $\rho (\xi _1) \le \rho (\xi _2)$ for $\xi _1,\xi _2 \in L^{\infty }_{+}(\Omega ,\mathcal {F},\mathbb {P})$ and $\xi _1 \le \xi _2$.

#### Proof

(i) $$\begin{aligned} \rho (\xi + c)&= \int _0^\infty w \left( \mathbb {P}(\xi + c \ge z)\right) dz \\&= \int _0^\infty w(\mathbb {P}(\xi \ge z - c))dz \\&= \int ^0_{-c} w \left( \mathbb {P}(\xi \ge z)\right) dz + \int _0^\infty w \left( \mathbb {P}(\xi \ge z)\right) dz\\&= c + \rho (\xi ) \end{aligned}$$
  Moreover, we have
  $$\begin{aligned} \rho (0)&= \int _0^\infty w(P(0 \ge z))dz\\&= \int _{\{0\}} w(P(0 \ge z))dz \\&= \int _{\{0\}} w(1)dz = 0 \end{aligned}$$
  Hence, by the first equality above, we have $\rho (0 + c) = c$ for $c \ge 0$.
(ii) $$\begin{aligned} \rho (\lambda \xi )&= \int _0^\infty w(\mathbb {P}(\lambda \xi \ge z))dz \\&= \lambda \int _0^\infty w\left( \mathbb {P}\left( \xi \ge \frac{z}{\lambda }\right) \right) d\frac{z}{\lambda }\\&= \lambda \rho (\xi ) \end{aligned}$$
(iii) Since $\xi _1 \le \xi _2$ and *w* is monotone, we have for any $z \ge 0$
  $$\begin{aligned} \mathbb {P}(\xi _1 \ge z)&\le \mathbb {P}(\xi _2 \ge z) \\ w(\mathbb {P}(\xi _1 \ge z))&\le w(\mathbb {P}(\xi _2 \ge z)) \end{aligned}$$
  Thus, we have $\rho (\xi _1) \le \rho (\xi _2)$. $\square$

### Dynamic probability distortion on stochastic processes

The main issue occurs when one tries to extend (2.1) to the multi-period setting. In particular, it is not clear what the “conditional version" of distortion operator is. Hence, first the corresponding operator for multi-temporal dynamic setting is constructed.

Fix $T \in \mathbb {N}_0$ and denote ${{\mathcal {T}}}\triangleq [0,1,\ldots , T]$ and $\tilde{{{\mathcal {T}}}}\triangleq [0,1,\ldots ,T-1]$. Let $\Omega$ be the sample space with its respective sigma algebra denoted by $\mathcal {F}$. Let $\mathcal {F}_0\subset \mathcal {F}_1 \subset \ldots \mathcal {F}_T\subset \mathcal {F}$ be a the filtration, and $\mathbb {P}$ being the probability measure on $\Omega$ such that $(\Omega ,\mathcal {F}, (\mathcal {F}_t)_{t\in {{\mathcal {T}}}}, \mathbb {P})$ is the stochastic basis. Let $\xi = (\xi _t)_{t \in {{\mathcal {T}}}}$ be a discrete time *non-negative* stochastic process that is adapted to the filtration $(\mathcal {F}_t)_{t \in {{\mathcal {T}}}}$ and uniformly bounded that is $\sup _{t\in {{\mathcal {T}}}}\hbox {ess}\, \hbox {sup}(\xi _t) < \infty$. We denote in that case $\xi \in L_{+}(\Omega ,(\mathcal {F}_t)_{t \in {{\mathcal {T}}}}, \mathbb {P})$. Then, if we define for $t \in {{\mathcal {T}}}$

$$\begin{aligned} \rho _t(\xi _T) \triangleq \int _0^\infty w \left( \mathbb {P}(\xi _T \ge z | \mathcal {F}_t)\right) dz, \end{aligned}$$

we do not necessarily have

$$\begin{aligned} \rho (\xi ) = \rho (\rho _t(\xi )) \end{aligned}$$

In particular, the “tower property” of expectation operator fails in distortion operators (see Example 2.1 below.). In the context of stochastic optimization, this implies that the optimization problem becomes “time-inconsistent”, i.e. the “Dynamic Programming Principle” (DPP) does not hold. On the other hand, for $w(x) = x$, the distortion operator (2.1) reduces to expectation operator, whereas for $\mathbb {E}_t[\xi _T] \triangleq \mathbb {E}[\xi _T|\mathcal {F}_t]$, we have $\mathbb {E}[\xi _T] = \mathbb {E}[\mathbb {E}_t[\xi _T]]$ with the towering property, and DPP holds.

Analogous to Definition 2.1, we define first *dynamic distortion mappings* on a filtered probability space $(\Omega , \mathcal {F}, (\mathcal {F}_t)_{t \in {{\mathcal {T}}}}, \mathbb {P})$ in a multitemporal setting as follows.

#### Definition 2.2

Let $t \in \tilde{{{\mathcal {T}}}}$ and $\xi _{t+1} \in L^{\infty }_{+}(\Omega ,\mathcal {F}_{t+1},\mathbb {P})$. Also consider the distortion function $w(\cdot )$ as in Definition 2.1.

- A one-step dynamic distortion mapping $\varrho _{t+1|t}: L^{\infty }_{+}(\Omega ,\mathcal {F}_{t+1},\mathbb {P}) \rightarrow L^{\infty }_{+}(\Omega ,\mathcal {F}_{t},\mathbb {P})$ is defined as
  $$\begin{aligned} \varrho _{t+1|t}(\xi _{t+1})&\triangleq \int _0^\infty w \left( \mathbb {P}(\xi _{t+1} \ge z_{t+1}| \mathcal {F}_{t})\right) dz_{t+1} \end{aligned}$$
- A mapping $\varrho _t : L^{\infty }_{+}(\Omega ,\mathcal {F}_{T},\mathbb {P}) \rightarrow L^{\infty }_{+}(\Omega ,\mathcal {F}_{t},\mathbb {P})$ is called a dynamic distortion mapping, if it is composition of one step dynamic distortion mappings of the form
  $$\begin{aligned} \varrho _t \triangleq \varrho _{t+1|t}\circ \ldots \circ \varrho _{T|T-1} \end{aligned}$$

#### Remark 2.2

Definition 2.2 is well defined. Indeed, let $\xi _T \in L^{\infty }_{+}(\Omega ,\mathcal {F}_{T},\mathbb {P})$, going backwards iteratively, by properties of *w* and construction of $\varrho _t$, uniform boundedness and $\mathcal {F}_s$ measurability at each $s \in [t,\ldots ,T]$ are preserved, such that $\varrho _t(\xi _T)$ maps $\xi _T$ to $L^{\infty }_{+}(\Omega ,\mathcal {F}_{t},\mathbb {P})$. Furthermore, by construction $\varrho _s(\cdot ) = \varrho _s(\varrho _t(\cdot ))$ for $0 \le s \le t \le T$. In particular, it is a time-consistent operator.

#### Lemma 2.3

For $t \in {{\mathcal {T}}}$, let $\varrho _t: L^{\infty }_{+}(\Omega ,\mathcal {F}_{T},\mathbb {P}) \rightarrow L^{\infty }_{+}(\Omega ,\mathcal {F}_{t},\mathbb {P})$ be the dynamic distortion operator as in Definition 2.2 and $\xi , \xi _1,\xi _2 \in L^{\infty }_{+}(\Omega ,\mathcal {F}_{T},\mathbb {P})$. Then

(i) $\varrho _t$ is positively translation invariant, i.e., $\varrho _t(\xi + c) = \varrho _t(\xi ) + c$ $\mathbb {P}$-a.s., if *c* is nonnegative and $\mathcal {F}_t$ measurable.
(ii) $\varrho _t$ is positively homogeneous, i.e. $\varrho _t(\lambda \xi ) = \lambda \varrho _t(\xi )$ $\mathbb {P}$-a.s. for any scalar $\lambda \ge 0$.
(iii) $\varrho _t$ is monotone, i.e. $\varrho _t(\xi _1) \le \varrho _t(\xi _2)$ $\mathbb {P}$-a.s. for $\xi _1 \le \xi _2$, $\mathbb {P}$-a.s..

#### Proof

The proof is a simple modification of Lemma 2.2. $\square$

Next, we illustrate the failure of towering property that causes time inconsistency via the following example.

#### Example 2.1

Let *X* and *Y* be two i.i.d. random variables on some probability space $(\Omega ,\mathcal {F},\mathbb {P})$ with $\mathbb {P}(X = 1) = \mathbb {P}(X = 2) = \frac{1}{2}$, $\mathbb {P}(Y = 1) = \mathbb {P}(Y = 2) = \frac{1}{2}$ and $w(x) = x ^{1/2}$. Let $\xi _1 = X$ and $\xi _2 = X + Y$, with $\mathcal {F}_1 = {\sigma }(X)$ and $\mathcal {F}_2 = {\sigma }(X,Y)$. Then

$$\begin{aligned} \rho _{2|1}(\xi _2)&= X + \rho _{1|0}(Y)\\&= X + w(1) + w\left( \frac{1}{2}\right) , \end{aligned}$$

where we use Lemma 2.3 i) in the first equality, above. Similarly, we have

$$\begin{aligned} \rho _{1|0} \circ \rho _{2|1}(\xi _2)&= \rho (X) + w(1) + w\left( \frac{1}{2}\right) \\&= 2w(1) + 2 w\left( \frac{1}{2}\right) \\&= 2 + 2\left( \frac{1}{2}\right) ^{1/2}. \end{aligned}$$

On the other hand, we have

$$\begin{aligned} \rho (\xi _2)&= \int _0^\infty w(P(\xi _2 \ge z))dz \\&= \int _{[0,2]} w(P(\xi _2 \ge z))dz + \int _{(2,3]} w(P(\xi _2 \ge z))dz + \int _{(3,4]} w(P(\xi _2 \ge z))dz\\&= 2 w(1) + \int _{(2,3]}w(3/4) dz + \int _{(3,4]}w(1/4) dz \\&= 2 + w(3/4) + w(1/4)\\&= 2 + \left( \frac{3}{4}\right) ^{1/2} + \left( \frac{1}{4}\right) ^{1/2} \end{aligned}$$

Hence, $\rho _{1|0}\circ \rho _{2|1}(\xi _2) > \rho (\xi _2)$ by strict concavity of *w*. We further note that the two expressions would be equal to each other, if $w(x) = x$.

## Controlled markov chain framework

In this section, we are going to introduce the necessary background on discrete-time controlled Markov processes (a.k.a. Markov decision processes (MDPs) (see e.g. Hernandez-Lerma and Lasserre 1996) that we are going to work, but using now the dynamic probability distortion framework.

We take the control model

$$\begin{aligned} \mathcal {M}_t := ( X_t, A_t, \mathbb {K}_t, Q_t, F, r_t) \end{aligned}$$

with the following components:

- $X_t$ and $A_t$ denote the state and action (or control) space, respectively, which are assumed to be Borel spaces, that is, Borel subsets of complete and separable metric spaces with their corresponding Borel ${\sigma }$-algebras $\mathcal {B}(X_t)$ and $\mathcal {B}(A_t)$.
- For each $x\in X_t$, let $A_t(x) \subset A_t$ be the set of all admissible controls in the state $x$. We assume that $A_t(x)$ is compact for $t \in {{\mathcal {T}}}$ and denote
  3.1 $$\begin{aligned} \mathbb {K}_t := \left\{ (x,a): x \in X_t,\; a \in A_t(x)\right\} \end{aligned}$$
  as the set of feasible state-action pairs.
- We define the system function as
  3.2 $$\begin{aligned} x_{t+1} \triangleq F_t(x_t, a_t, \eta _t) \end{aligned}$$
  for all $t \in \tilde{{{\mathcal {T}}}}$ with $x_t \in X_t$ and $a_t \in A_t$, and i.i.d. random variables $(\eta _t)_{t \in \tilde{{{\mathcal {T}}}}}$ on a probability space $(Y,\mathcal {B}(Y), P^\eta )$ with values in *Y* that are complete separable Borel spaces. We assume that the mapping $(s,x,a) \rightarrow F(s,x,a,y)$ in (3.2) is continuous on $S_t \times X_t \times A_t$ for every $y \in Y$ at every $t \in \tilde{{{\mathcal {T}}}}$.
- Let
  $$\begin{aligned} \Omega \triangleq \otimes ^T_{t = 0} X_t\end{aligned}$$
  and for $t \in {{\mathcal {T}}}$, and
  $$\begin{aligned} \mathcal {F}_t&= {\sigma }\left( X_0, A_0, \ldots , X_{t-1},A_{t-1},X_t \right) \end{aligned}$$
  be the filtration of increasing ${\sigma }$-algebras.
- Let $\mathbb {F}_t$ be the family of measurable functions and $\pi _t \in \mathbb {F}_t$ with $\pi _t: X_t\rightarrow A_t$ for $t \in \tilde{{{\mathcal {T}}}}$. A sequence $( \pi _t )_{t\in \tilde{{{\mathcal {T}}}}}$ of functions $\pi _t \in \mathbb {F}_t$ is called an admissible control policy (or simply a policy), and the function $\pi _t(\cdot )$ is called the decision rule or control at time *t*. We denote by $\Pi$ the set of all admissible control policies.
- Let $r_t(x_t,a_t): X_t \times A_t \rightarrow \mathbb {R}_{+}$ for $t \in \tilde{{{\mathcal {T}}}}$ and $r_T: X_T \rightarrow \mathbb {R}_{+}$ be the non-negative real-valued reward-per-stage and terminal reward function, respectively. For $(\pi _t)_{t \in \tilde{{{\mathcal {T}}}}} \in \Pi$, we write
  $$\begin{aligned} r_t(x_t,\pi _t)&\triangleq r_t(x_t,\pi _t(x_t))\\&\triangleq r_t(x_t,a_t). \end{aligned}$$
- Let $\pi \in \Pi$ and $x_0 \in X_0$ be given. Then, there exists a unique probability measure $\mathbb {P}^\pi$ on $(\Omega , \mathcal {F})$ such that, given $x \in X_t$, a measurable set $B_{t+1} \subset X_{t+1}$ and $(x_t,a_t) \in \mathbb {K}_t$, for any $t \in \tilde{{{\mathcal {T}}}}$, we have
  $$\begin{aligned} Q_{t+1}(B_{t+1} | x_t,a_t) \triangleq \mathbb {P}^{\pi }_{t+1} (x_{t+1} \in B_{t+1}|x_t,a_t,\ldots ,x_0). \end{aligned}$$
  Here, $Q_{t+1}(B_{t+1}|x_t,a_t)$ is the stochastic kernel (see e.g. Hernandez-Lerma and Lasserre 1996). Namely, for each pair $(x_t,a_t) \in \mathbb {K}_t$, $Q_{t+1}(\cdot |x_t,a_t)$ is a probability measure on $X_{t+1}$, and for each $B_{t+1} \in \mathcal {B}_{t+1}(X_{t+1})$, $Q_{t+1}(B_{t+1}|\cdot ,\cdot )$ is a measurable function on $\mathbb {K}_t$. We remark that at each $t\in {{\mathcal {T}}}$, the stochastic kernel depends only on $(x_t,a_t)$ rather than the whole history $(x_0,a_0,x_1,a_1,\ldots ,a_t,x_t)$. By (3.2), we have
  $$\begin{aligned} Q_{t+1}(B_{t+1}|x_t,a_t) = \int _Y I_{B_{t+1}}[F(x_t,a_t,y)] dP^\eta (y),\;B_{t+1} \in \mathcal {B}(X_{t+1}), \end{aligned}$$
  where $I_{B_{t+1}}$ denotes the indicator function of $B_{t+1}$.

### Assumption 3.1

- The reward functions $r_t(x_t,a_t)$ for $t \in \tilde{{{\mathcal {T}}}}$ and $r_T(x_T)$ are nonnegative, continuous in their arguments and uniformly bounded i.e. $0 \le r_t(x_t,a_t) < \infty$ and $0 \le r_T(x_T) < \infty$.
- The multi-function (also known as a correspondence or point-to-set function) $x \rightarrow A_t(x)$ is upper semi-continuous (u.s.c.). That is, if $\{x^m\} \subset X_t$ and $\{ a^m\} \subset A_t(x^m)$ are sequences such that $x^m \rightarrow \bar{x}$, and $a^m \rightarrow \bar{a}$, then $\bar{a} \in A_t(\bar{x})$ for $t \in \tilde{{{\mathcal {T}}}}$.
- For every state $x \in X_t$, the admissible action set $A_t(x)$ is compact for $t \in \tilde{{{\mathcal {T}}}}$.

## Optimal control problem

### Main Result

For every $t \in \tilde{{{\mathcal {T}}}}$, $x_t \in X_t$ and $\pi \in \Pi$, let

$$\begin{aligned} V_t(x_t,\pi )&\triangleq \varrho _t\left( \sum ^{T-1}_{i=t} r_i(x_i,\pi _i) + r_T(x_T)\right) \end{aligned}$$

be the performance evaluation from time $t\in \tilde{{{\mathcal {T}}}}$ onwards using the policy $\pi \in \Pi$ given the initial condition $x \in X_t$. The corresponding optimal (i.e. maximal) value is then

4.1 $$\begin{aligned} V^*_t(x_t)&\triangleq \sup _{\pi \in \Pi } V_t(x_t,\pi ) \end{aligned}$$

A control policy $\pi ^* = (\pi ^*_t)_{t \in \tilde{{{\mathcal {T}}}}}$ is said to be optimal if it attains the maximum in (4.1), that is

4.2 $$\begin{aligned} V^*_t(x)&= V_t(x,\pi ^*)\; \text {for all } x\in X_t\;\text {and for } t \in \tilde{{{\mathcal {T}}}}. \end{aligned}$$

Thus, the optimal control problem is to find an optimal policy and the associated optimal value (4.2) for all $t \in {{\mathcal {T}}}$. We now present the main result of the paper.

#### Theorem 4.1

The optimization problem (4.1) obeys dynamic programming principle and has an optimal policy $\pi ^* \in \Pi$. Furthermore, $V^*_t(x_t)$ is continuous in its argument.

### Proof of Theorem 4.1

To prove Theorem 4.1, we need the following key lemma.

#### Lemma 4.1

Let $\mathbb {K}$ be defined as in (3.1). Let $V: \mathbb {K}\rightarrow \mathbb {R}$ be a nonnegative continuous function. For $x_t \in X_t$, define

$$\begin{aligned} V^*(x_t) \triangleq \sup _{a \in A} V(x_t,a). \end{aligned}$$

Then, for any $x_t \in X_t$, there exists a $\mathcal {B}(X_t)$ measurable mapping $\pi ^*_t: X \rightarrow A$ such that

4.3 $$\begin{aligned} V^*(x_t) = V(x_t,\pi ^*_t(x_t)) \end{aligned}$$

and $V^*:X_t \rightarrow \mathbb {R}$ is continuous.

#### Proof

By Lemma 4.1 in Rieder (1978), there exists $\mathcal {B}( X_t)$ measurable mapping $\pi ^*: X_t \rightarrow A_t$ such that (4.3) holds and $V^*(x_t)$ is upper semi-continuous. But, since $V(\cdot ,\cdot )$ is continuous, $\sup _{a\in A} V(x_t,a)$ is lower semi-continuous in *x*, as well. Hence, $V^*(\cdot ,\cdot )$ is continuous in its arguments. $\square$

#### Lemma 4.2

Suppose Assumption 3.1 holds true. Then, supremum is attained at (4.1) for some $\mathcal {B}(X_t)$ measurable mapping $\pi ^*_t(x_t) = a^*_t$ for $t \in \tilde{{{\mathcal {T}}}}$. Furthermore, each $V^*_t$ is continuous.

#### Proof

We will show only the case for $t = T-1$. The others follow going backwards iterative down to $t = 0$. We first show that

$$\begin{aligned} (x_{T-1},a_{T-1}) \rightarrow \int _0^\infty w \left( \mathbb {P}^{\pi } (r_T(F(s_{T-1}, x_{T-1},a_{T-1},\eta _{T-1})) \ge z_T \right) dz_T \end{aligned}$$

is continuous in its arguments. Let $(x^m_{T-1},a^m_{T-1}) \rightarrow (x_{T-1},a_{T-1})$ as $m \rightarrow \infty$. Then, we have

$$\begin{aligned}&\lim _{m\rightarrow \infty } V_{T-1} (x^m_{T-1},a^m_{T-1}) \\&\quad = \lim _{m\rightarrow \infty } \int _0^\infty w \left( \mathbb {P}^{\pi } ( r_T(F(x^m_{T-1},a^m_{T-1},\eta _{T-1})) \ge z_T \right) dz_T \\&\quad = \int _0^\infty \lim _{m\rightarrow \infty } w \left( \mathbb {P}^{\pi } (r_T(F(x^m_{T-1},a^m_{T-1},\eta _{T-1})) \ge z_T \right) dz_T \\&\quad = \int _0^\infty w \left( \lim _{m\rightarrow \infty } \mathbb {P}^{\pi } (r_T(F(x^m_{T-1},a^m_{T-1},\eta _{T-1})) \ge z_T \right) dz_T\\&\quad = \int _0^\infty w \left( \mathbb {P}^{\pi }( \lim _{m\rightarrow \infty } r_T(F(x^m_{T-1},a^m_{T-1},\eta _{T-1})) \ge z_T \right) dz_T \\&\quad = \int _0^\infty w \left( \mathbb {P}^{\pi }( r_T(F(x_{T-1},a_{T-1},\eta _{T-1})) \ge z_T \right) dz_T \end{aligned}$$

The second equality follows by boundedness of $r_T()$, $w(\cdot )$ and Lebesgue dominated convergence theorem. The third equality follows by continuity of $w(\cdot )$, the fourth equality follows by continuity of probability measure, and the fifth equality follows by continuity of transition $F(\cdot ,\cdot ,\cdot )$ as in (3.2). Hence, $V_{T-1}(\cdot ,\cdot )$ is continuous in its arguments. The result follows by Lemma 4.1. $\square$

Now, we are ready to prove Theorem 4.1.

#### Proof of Theorem 4.1

We have

$$\begin{aligned}&V^*_{T-1}( x_{T-1})\\&\qquad {}= \sup _{a_{T-1} \in A_{T-1}(x_{T-1})} \int _0^\infty w \left( \mathbb {P}^{\pi } (r_T( F(x_{T-1}, a_{T-1}),\eta _{T-1} )) \ge z_T \right) dz_T\\&\qquad {}= \int _0^\infty w \left( \mathbb {P}^{\pi } (r_T( F(x_{T-1}, \pi ^*_{T-1}(s_{T-1},x_{T-1}),\eta _{T-1} )) \ge z_T \right) dz_T. \end{aligned}$$

By Lemma (4.2), there exists a $\mathcal {B}(X_{T-1})$ measurable mapping $\pi _{T-1} \in \mathbb {F}_{T-1}$ such that $\pi _{T-1}^*(x_{T-1}) = a^*_{T-1}$, and $V^*_{T-1}(x_{T-1})$ is continuous. Hence, using Lemma 2.2(i) for $t = T-2$, we have

$$\begin{aligned}&V^*_{T-2}(x_{T-2}) \\&\qquad {}= \sup _{\begin{array}{c} a_{T-2} \in A_{T-2}(x_{T-2})\\ a_{T-1} \in A_{T-1}(x_{T-1}) \end{array}} \left\{ \int _0^\infty w ( \mathbb {P}^{\pi }( r_{T-2}(x_{T-2},a_{T-2}) \right. \\&\qquad {}\qquad {}\left. +\, V^*_{T-1} (F(x_{T-2},a_{T-2},\eta _{T-2})) \ge z_{T-1}) dz_{T-1}\right\} \end{aligned}$$

By Lemma (4.2) again, it admits an optimal policy $a^*_{T-2} \in A_{T-2}$ such that

$$\begin{aligned}&V^*_{T-2}(x_{T-2}) \\&\qquad {}= \left\{ \int _0^\infty w ( \mathbb {P}^{\pi }( r_{T-2}(x_{T-2},a^*_{T-2}) \right. \\&\qquad {}\qquad {}\left. +\, V^*_{T-1} (F(x_{T-2},a^*_{T-2},\eta _{T-2})) \ge z_{T-1}) dz_{T-1}\right\} \end{aligned}$$

Going backwards iterative, we conclude that dynamic programming holds, (4.1) admits an optimal policy $\pi ^* \in \Pi$ attaining supremum that depends only on $s_t$ and on $x_t$ at each $t \in \tilde{{{\mathcal {T}}}}$. Furthermore, $V^*_{t}(\cdot )$ is continuous again by Lemma (4.2). Hence, we conclude the proof.

Based on our main result, the methodology is as follows. Given the distortion operator along with the controlled Markov process, one checks whether the framework with reward and control sets satisfy the requirements in 3.1, Theorem 4.1 reveals that the dynamic programming can be applied to find the optimal controls along with the optimal value function. The next section exemplifies this.

## An application to portfolio optimization

### Model

Suppose an investor has a portfolio of *n* stocks. The prices of *n* stocks at $t \in {{\mathcal {T}}}$ are denoted by

$$\begin{aligned} S_t \triangleq (S^1_t,\ldots ,S^n_t). \end{aligned}$$

The price of stock $i \in [1,2,,\ldots ,n]$ at time $t \in \tilde{{{\mathcal {T}}}}$, denoted by $S^i_t$, has dynamics

5.1 $$\begin{aligned} S^i_{t+1} = (1 + r^i) S^i_t \text { with probability } p^i, \end{aligned}$$

where $-1< r^i < 1$ is the proportional return rate of price of *i*th stock $S^i_{t}$. Let $P^\eta (\cdot )$ denote the joint probability mass function of $S_t$ for $t \in {{\mathcal {T}}}$. Let $\pi = (\pi _t)_{t \in {{\mathcal {T}}}}$ be the policy of the investor that stands for the number of shares of *n* stocks investor is holding at time $t \in \tilde{{{\mathcal {T}}}}$ with

$$\begin{aligned}&\pi _t: S_t \rightarrow \mathbb {R}^n, \end{aligned}$$

where $\pi _t$ is $\mathcal {B}(S_t)$ measurable. We assume that the investor has a capacity to be in the long or short position. Namely, we take that $\Vert \pi _t(x)\Vert \le C$ for some $C > 0$, for all $x \in S_t$ and $t \in \tilde{{{\mathcal {T}}}}$. We denote by $\Pi$ the admissible strategies $(\pi _t)_{t \in \tilde{{{\mathcal {T}}}}}$ that are $\mathcal {B}(S_t)$ measurable and uniformly bounded by *C*. We take that the market is self-financing in the sense

$$\begin{aligned} Y_{t+1} = \pi ^\intercal _{t}S_{t+1} \text { for } t \in \tilde{{{\mathcal {T}}}}, \end{aligned}$$

with $Y_{t+1}$ being the value of the portfolio and $S_{t+1}$ being the *n*-dimensional vector as defined in (5.1) at time $t+1$ such that denoting $x_{-1} \triangleq x_0$,

$$\begin{aligned} \Delta Y_{t}&\triangleq Y_{t} - y_{t-1}, \end{aligned}$$

is the difference of the total wealth between time *t* and $t-1$ for $t\in {{\mathcal {T}}}$. Hence, the reward function at $t \in {{\mathcal {T}}}$ reads as

$$\begin{aligned}&r_{t}(s_{t-1},s_t,\pi _t) = \Delta Y_t\\&r_{T}(s_{T-1},s_T) = \Delta Y_T\\&Y_{t} = y_{t-1} + r_t(s_{t-1},s_t,\pi _t), \end{aligned}$$

Let $w(x) = x^2$ for $x \in [0,1]$ be the distortion of the probability function such that for a fixed $\pi _ {T-1}$ given $Y_{T-1} = y_{T-1}$ and $S_{T-1} = s_{T-1}$, the performance measure is defined by

$$\begin{aligned} \varrho _{T-1}(Y_T)&\triangleq \int _0^\infty \bigg (\mathbb {P}^\pi (y_{T-1} + \pi ^\intercal _{T-1}( S_{T} - s_{T-1}) \ge z_{T} | y_{T-1}, s_{T-1}) \bigg )^2 dz_T\\&= y_{T-1} + \int _0^\infty \bigg (\mathbb {P}^\pi (\pi ^\intercal _{T-1}( S_{T} - s_{T-1}) \ge z_{T} | y_{T-1}, s_{T-1}) \bigg )^2 dz_T\\&= V_{T-1}(y_{T-1},s_{T-1},\pi ) \end{aligned}$$

such that

$$\begin{aligned} V^*_{T-1}(y_{T-1},s_{T-1}) = \max _{\pi \in \Pi } V_{T-1}(y_{T-1},s_{T-1},\pi ). \end{aligned}$$

Here, in the second equality Lemma 2.3 is used. Hence, going backwards iterative, we have at each time $t \in \tilde{{{\mathcal {T}}}}$

5.2 $$\begin{aligned} V_t(y_t,s_t,\pi )&= y_t + \int _0^\infty \bigg ( \mathbb {P}^\pi ( V_{t+1}(Y_{t+1},S_{t+1},\pi )\ge z_{t+1} | y_t,s_t) \bigg )^2dz_{t+1}\nonumber \\ V^*_t(y_t,s_t)&= \max _{\pi \in \Pi } V_t(y_t,s_t,\pi ). \end{aligned}$$

The methodology is that the application satisfies Theorem 4.1. Indeed, it is immediate to see that the conditions for Assumption 3.1 are satisfied for the reward function and the action sets. Thus, Theorem 4.1 can be applied. In particular, Theorem 4.1 allows the dynamic programming yielding an optimal strategy $(\pi ^*_t)_{t \in \tilde{{{\mathcal {T}}}}} \in \Pi$ attaining (5.2) along with the optimal value $V^*_t$ at each time $t \in \tilde{{{\mathcal {T}}}}$.

### Numerical example

Consider discrete time set ${{\mathcal {T}}}= \{0,1,2\}$ and two stocks Stock A and Stock B. The prices of Stock A and Stock B at $t \in {{\mathcal {T}}}$ are denoted by $S_t^A$ and $S_t^B$ respectively. Prices at $t = 0$ are denoted by $S_0^A = S_0^B = 1$. Additionally, suppose that an investor can buy one of Stock A, Stock B or portfolio Stock AB with equal shares of both stocks. His initial wealth to be invested equals 1. An investor can switch between Stock A, Stock B, or trade-off between stocks to share equally current wealth into both stocks at each time $t=0,1,2$. The current state of wealth changes as stock price goes up or down with movement rate denoted by $r^A$ and $r^B$, respectively. They are independent random variables satisfying

$$\begin{aligned} \mathbb {P}(r^A = 0.22)&= \mathbb {P}(r^A = -\,0.20) = \frac{1}{2}\\ \mathbb {P}(r^B = 0.12)&= \mathbb {P}(r^B = -\,0.10) = \frac{1}{2}. \end{aligned}$$

In particular, the expected value of return for the given share is positive $\mathbb {E}[r^A] = \mathbb {E}[r^B] = \mathbb {E}[0.5r^A+0.5r^B] = 0.01$. Hence, 2-period investment, in the same stock, as well as investing in equal shares portfolio are expected to give return of $1.01^2-1 = 0.0201$. The distortion function $w(x) = x^2$ on $x \in [0,1]$. Hence, $w(\mathbb {P}(r^A=0.22)) = (\frac{1}{2})^2=\frac{1}{4}$ and $w(\mathbb {P}(r^A=-0.20)) =1- (\frac{1}{2})^2=\frac{3}{4}$. The highest return using the distortion operator at $t=0$ equals

5.3 $$\begin{aligned} \varrho _{1|0}^B=0.912>\varrho _{1|0}^{AB}=0,902>\varrho _{1|0}^A= 0.864. \end{aligned}$$

Here $\varrho _{1|0}^A$ denotes the value if the investor starts with putting all his wealth into *A*. $\varrho ^B_{1|0}$ and $\varrho ^{AB}_{1|0}$ denote accordingly. By (5.3), optimal strategy is to invest in Stock B for $t=1$ and continuing this investment for $t=2$. It is depicted in Fig. 2, below. However, return estimated with dynamic probability distortion is $0.912-1=-0.088$. In particular, it is negative.

**Fig. 1:**
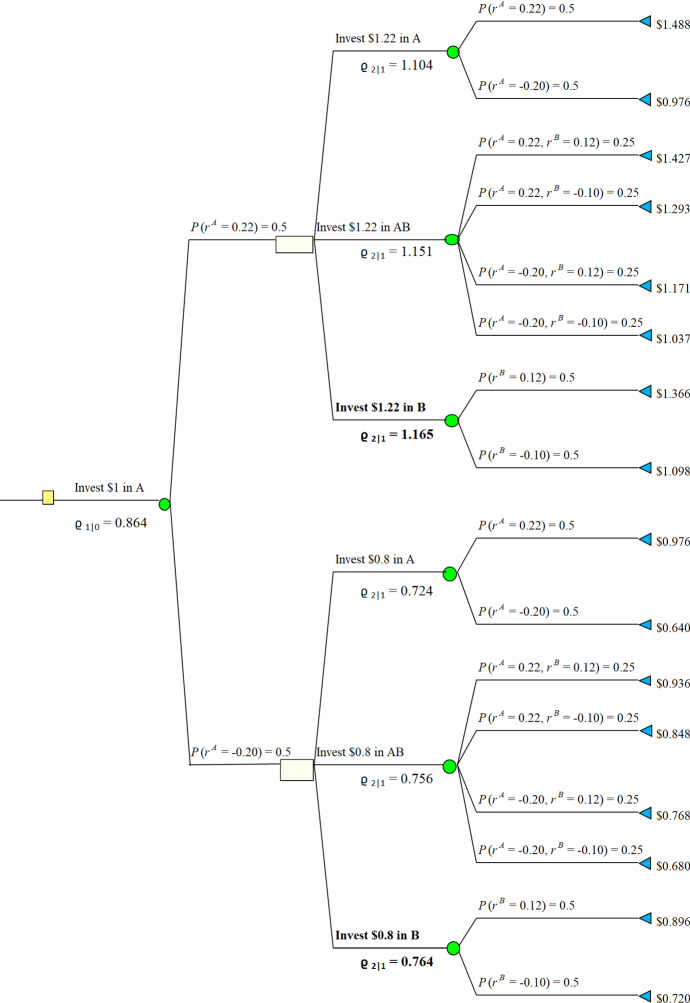
Starting with Investing in Stock *A*

**Fig. 2:**
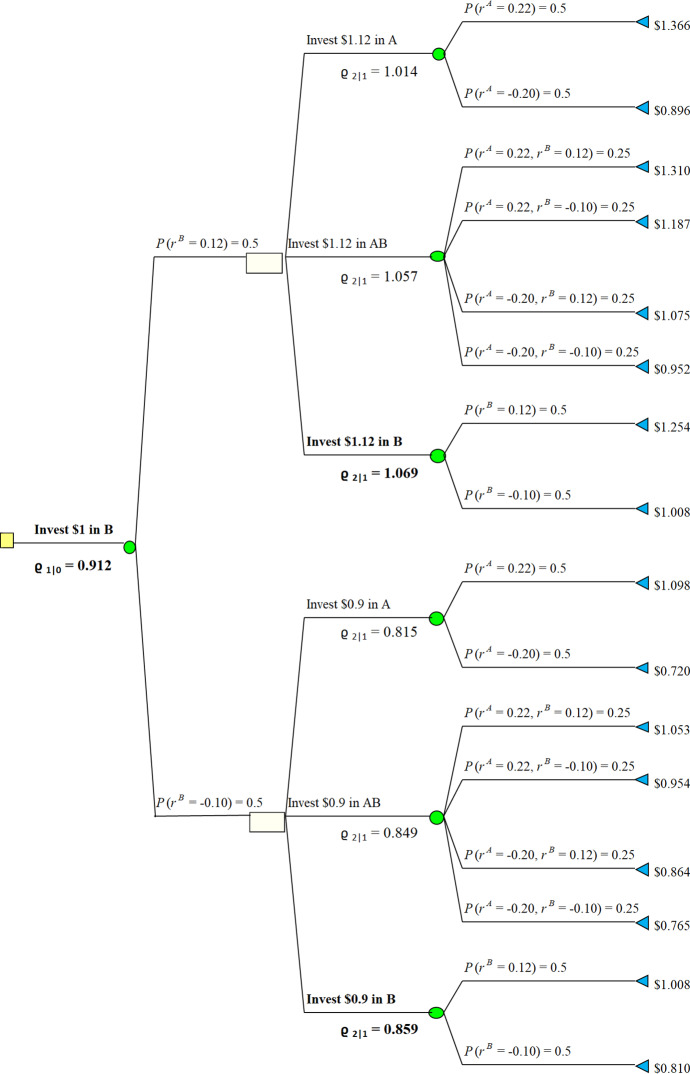
Starting with Investing in Stock *B*

**Fig. 3:**
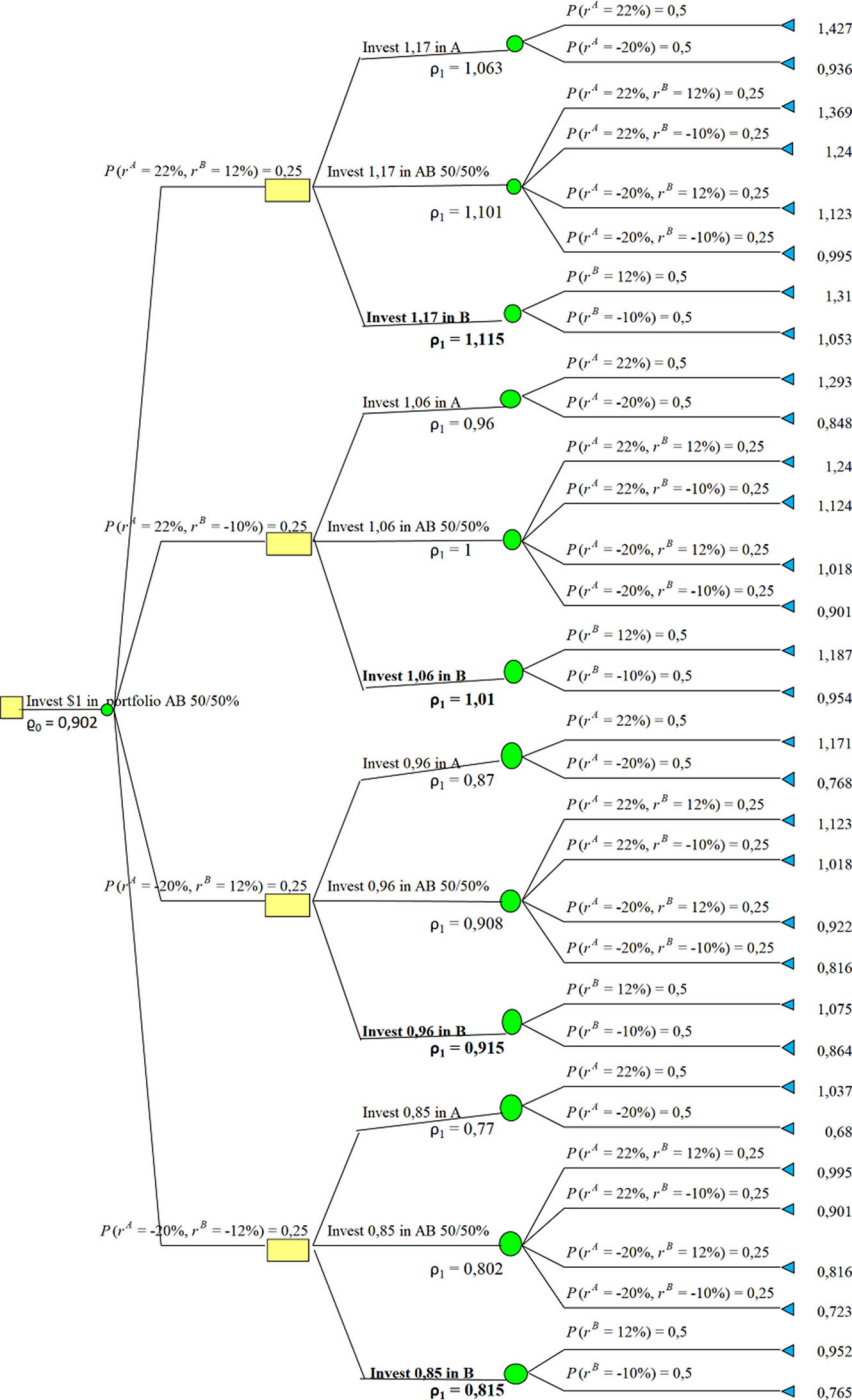
Starting with Investing in Stock *AB*

Moreover, Fig. 1 shows that if one starts with investing in Stock A at $t=0$, then the optimal decision is to switch to Stock B at $t=1$. In this case the dynamic operator gives value $\varrho ^B_0=0.864$, and return at $t=2$ is $0.864-1=-0.136$. Similarly, we see at Fig. 3 that if one starts with investing equal shares into Stock A and Stock B at $t=0$, then the investor should switch to Stock B, and the distortion operator gives $\varrho ^{AB}_0=0.902$. The results at Figs. 1, 2 and 3 reveal that the distortion operator prevents an investor from taking risk by investing in Stock A even though the expected rate of return is equal in any period of time. The investor chooses Stock B only irrelevant of his first stake at $t=0$. Furthermore, the strategy is time consistent. The Figs. 1, 2 and 3 summarize the corresponding actions, where the bold fonts at each time epoch denote the optimal actions.

## Conclusion and future work

This paper discusses assessing multi-period risk of an investment using distortion operators. The peculiarity of these operators lies in the fact that they give optimum controls that are not time consistent in a multi-period framework. The dynamic distortion operator introduced in this paper is the way of multi-period aggregation of the distortion operator that satisfies time consistency and hence dynamic programming principle. Furthermore, it is demonstrated how these operators work and apply to the investment problem. The main economic implication of the presented methodology is as follows. If observed probabilities are distorted but observed outcomes of stock return rate are viable, then the dynamic distortion operators produce optimum multi-period investment strategy that is robust to risk and is return effective.

Further research will be to focus on empirical results of investment strategies based on distortion operators. These results should be compared with alternative strategies using the same set of empirical data. For instance, the proposed framework can be compared with the strategies presented in Elze (2012); Majewski et al. (2020), which outperforms market return-risk efficiency. A scope of downside risk measures and models that are alternatives to the presented investing optimization methodology were proposed and applied by Barro and Canestrelli (2014). Also using second order stochastic dominance (SSD) relations (Batabyal and Killinis 2021) and convex risk measures are alternatives (Ruszczynski 2010; Follmer and Penner 2006; Fritelli et al. 2002) to the proposed methodology in this work, whose performance on empirical data is to be compared.
